# Value of Prophylactic Postoperative Antibiotic Therapy after Bimaxillary Orthognathic Surgery: A Clinical Trial

**Published:** 2014-10

**Authors:** Majid Eshghpour, Amin Khajavi, Mahmoud Bagheri, Elham Banihashemi

**Affiliations:** 1 *Dental Research Center, Mashhad University of Medical Sciences, Mashhad, Iran.*; 2 **General Practitioner, Mashhad, Iran.**

**Keywords:** Amoxicillin, Antibiotic therapy, Orthognathic surgery, Post-operative wound infection

## Abstract

**Introduction:**

Antibiotic therapy before or after orthognathic surgery is commonly recommended by surgeons to minimize the risk of wound infection. This article evaluates the value of Prophylactic antibiotic therapy in order to diminish the incidence of postoperative wound infection after orthognathic surgery.

**Materials and Methods:**

Fifty candidates for bimaxillary orthognathic surgery were divided into cases and controls. Cefazolin (1g) was administered intravenously to all participants 30 mins prior to surgery followed by a similar dose 4 hours later. Case-group patients ingested amoxicillin (500 mg) orally for 7 days after surgery. Postoperative wound infection was assessed using clinical features, and the P-value significance was set at P<0.05.

**Results:**

Both groups were similar according to gender, age, and operating time. During the follow-up period no infection was observed in either the case or control group.

**Conclusion:**

The results of this study suggest that long-term postoperative antibiotic therapy is not essential for the prevention of postoperative infection, and that application of aseptic surgical technique and hygiene instruction after surgery are sufficient.

## Introduction

The initial aim of orthognathic surgery is to reestablish the proper relationship between the maxilla and the mandible in order to provide improvements in speech, mastication, and esthetics (1). Since these operations are classified as "clean contaminated" procedures due to the incision through a mucosal surface which allows the entrance of oral endogenous flora, there is a 10–15% risk of wound infection (1). This risk might be minimized by application of an aseptic surgical technique and use of prophylactic antibiotics, although prescribing of postoperative antibiotic regimes remains a controversial concept (2). A systematic review on the effectiveness of postoperative antibiotic therapy after orthognathic surgery supported its value in decreasing the risk of wound infection (2), while in another study, prolonged postoperative antibiotic therapy was shown to be unnecessary in the absence of any other factors after orthognathic surgery (3). It has been also revealed that intravenous administration of postoperative antibiotics after orthognathic surgeries is as effective as oral administration in decreasing the rate of infection (4). 

On the other hand, there are articles that criticize the practice of prescribing antibiotics prophylactically to control postoperative infections (5,6), since antibiotic deployment could lead to the development of super infections (infections resistant to antibiotics). This concern highlights the need for further studies to determine the value of antibiotic prophylaxis with respect to postoperative infections following orthognathic surgery (3). 

This clinical trial was conducted in order to evaluate the value of 7 days of oral amoxicillin therapy in reducing the rate of postoperative infection in patients undergoing bimaxillary orthognathic surgeries.

## Materials and Methods

This clinical trial was performed among patients who were about to undergo bimaxillary orthognathic surgery at the Oral and Maxillofacial Department of Mashhad Dental School. The study protocol was approved by the ethics committee of Mashhad Medical Science University, and written testimonials were signed by all participants. Patients with exposure to antibiotics within a month prior to surgery and those with a history of allergic reaction to penicillin or cefazolin were excluded from the study. Moreover, immune-compromised patients, patients with acute or chronic sinusoidal problems, and those with a history of previous orthognathic surgery were also excluded. All surgery was performed by one surgeon, while another practitioner assessed the patients through the follow-up period. All operations involved both jaws and the repositioning of cut pieces in different alignments. Maxilla Le Fort I osteotomy or mandible bilateral sagittal split osteotomy (BSSO) was performed in all cases. 

Elastics were bonded directly after surgery to provide inter-maxillary fixation in all participants; none of the patients required inter-maxillary fixation with a hard wire. Rigid internal fixation was performed using mini plates and 2-mm screws. Fixation of maxillary pieces was achieved using four titanium plates, while three screws on each side were used in the mandible. 

A total of 50 patients were randomly divided into case and control groups (25 in each group). All patients received cefazolin (1 g single dose) intravenously 30 min prior to surgery, and the same dose was prescribed 4 hours after the first injection. In order to reduce the bacterial load of the oral cavity, all patients were asked to brush their teeth and use chlorhexidine mouth wash directly before surgery. Before suturing the incisions, sites were irrigated using normal saline (150 ml). 0.2% Chlorhexidine oral rinse was used by all patients twice a day for 2 weeks. A prescription of amoxicillin syrup (500 mg) was given to patients in the case group in addition to the antibiotic regime described above, while the control group was given placebo orally every 8 hours for a total of 1 week. Participants were assessed throughout the 3 days following surgery and were reexamined on a weekly basis for 6 weeks after discharge. 

Postoperative infection was diagnosed by a clinician after consideration of several variables including facial swelling, purulent discharge from the incision site, drainage, wound dehiscence, pain, or erythema. Total leukocyte count and erythrocyte sedimenta- tion rates were not considered as diagnostic variables for postoperative infection because a previous study showed that these values increase inevitably after surgery in all patients (1). After data collection, variables such as gender were compared using the *χ*^2^ test while the t-test was used for comparison of age and operation time. Further, the rates of infection between the two groups were compared using the *χ*^2^ corrected test. SPSS (version14.0) was used for statistical analysis and significance rate was set at P<0.05. 

## Results

Fifty patients (33 female and 17 male) were included in this study and were divided into case and control groups (25 patients in both). Different types of deformities were seen in the patients; 33 patients had skeletal CL III, 12 had skeletal CL II, and five cases had anterior open bite. Bimaxillary surgeries were performed in all patients and all were referred to the department during the follow-up period. The control group included 18 female and seven male patients, whereas the case group consisted of 15 female and 10 males. The mean age of the patients was 27 years (range, 17–35 years). No significant difference between the two groups was identified with regards to gender (P=0.8) or age (P=0.7). The duration of the operation ranged from 147 to 215 minutes and both groups were comparable with regards to this variable (P=0.3). No signs of postoperative infection were observed in the case or control groups, indicating no statistical difference in the incidence of postoperative wound infection between the two groups (P=1). 

## Discussion

In the current study, we compared the incidence of postoperative infection between patients who used 500 mg amoxicillin syrup for 7 days after orthognathic surgery and a control group. There was no significant difference in the rate of postoperative infection between the two groups suggesting that a single dose of cefazolin (1g) preoperatively and continued posto- peratively may be sufficient to minimize the risk of postoperative wound infections after orthognathic surgery. This indicates that prescribing long-term antibiotic therapy may not be necessary. 

Due to the side effects of antibiotics such as allergic reactions, toxic reactions, and secondary infections (1), prescribing an optimum dose of antibiotics is essential to prevent postoperative infections. However, as suggested by several studies, in the absence of primary infections, postoperative antibiotics may lead to the development of resistant strains; suggesting antibiotic treatments should not be used to control this kind of infection (5,6). Among the studies that support the necessity of antibiotic therapy in orthognathic surgeries, different antibiotic regimes have been proposed (1,2,7-12). For instance, in a 2011 systematic review, a single dose regime was recommended to control postoperative infections (8), while a meta-analysis revealed that extended postoperative antibiotic treatments have a place in reducing the risk of wound infections after orthognathic surgeries (2). Similar to the present study, it has been reported that there is no difference between high dosage administration of postoperative antibiotic therapy and a 7–10-day regime after orthognathic surgery (11), regarding postoperative infections. It has also been reported that although there is no significant difference between short-term and long-term prophylactic regimes with regards to reduction of postoperative infection rate, administration of long-term antibiotic therapy may reduce the morbidity of the operation (7).

## Conclusion

According to the results of this study, we conclude that the application of aseptic surgical technique and hygiene instructions after a surgery is more important than the use of long-term antibiotic therapy to prevent postoperative infections. Although the results of the present study may disprove the necessity of long-term postoperative antibiotic therapy following orthognathic surgeries, due to the small size of sample and insufficient exposure, further studies are recommended to prove the inessential role of long-term postoperative antibiotic therapy in decreasing the risk of wound infection following orthognathic surgeries. 

## References

[1] Zijderveld SA, Smeele LE, Kostense PJ, Tuinzing DB (1999). Preoperative antibiotic prophylaxis in orthognathic surgery: a randomized, double-blind, and placebo-controlled clinical study. Journal of Oral and Maxillofacial Surgery.

[2] Danda AK, Ravi P (2011). Effectiveness of postoperative antibiotics in orthognathic surgery: a meta-analysis. Journal of Oral and Maxillofacial Surgery.

[3] Kang SH, Yoo JH, Yi CK (2009). The efficacy of postoperative prophylactic antibiotics in orthognathic surgery: a prospective study in Le Fort I osteotomy and bilateral intraoral vertical ramus osteotomy. Yonsei Medical Journal.

[4] Tan SK, Lo J, Zwahlen RA (2011). Are postoperative intravenous antibiotics necessary after bimaxillary orthognathic surgery? A prospective, randomized, double-blind, placebo-controlled clinical trial. International Journal of Oral and Maxillofacial Surgery.

[5] Yrastorza JA (1976). Indications for antibiotics in orthognathic surgery. Journal of Oral Surgery.

[6] Peterson LJ, Booth DF (1976). Efficacy of antibiotic prophylaxis in intraoral orthognathic surgery. Journal of Oral Surgery.

[7] Baqain ZH, Hyde N, Patrikidou A, Harris M (2004). Antibiotic prophylaxis for orthognathic surgery: a prospective, randomised clinical trial. British Journal of Oral & Maxillofacial Surgery.

[8] Tan SK, Lo J, Zwahlen RA (2011). Perioperative antibiotic prophylaxis in orthognathic surgery: a systematic review and meta-analysis of clinical trials. Oral Surgery, Oral Medicine, Oral Pathology, Oral Radiology, and Endodontics.

[9] Chow LK, Singh B, Chiu WK, Samman N (2007). Prevalence of postoperative complications after orthognathic surgery: a 15-year review. Journal of Oral and Maxillofacial Surgery.

[10] Lindeboom JA, Baas EM, Kroon FH (2003). Prophylactic single-dose administration of 600 mg clindamycin versus 4-time administration of 600 mg clindamycin in orthognathic surgery: A prospective randomized study in bilateral mandibular sagittal ramus osteotomies. Oral Surgery, Oral Medicine, Oral Pathology, Oral Radiology, and Endodontics.

[11] Fridrich KL, Partnoy BE, Zeitler DL (1994). Prospective analysis of antibiotic prophylaxis for orthognathic surgery. International Journal of Adult Orthodontics and Orthognathic Surgery.

[12] Danda AK, Wahab A, Narayanan V, Siddareddi A (2010). Single-dose versus single-day antibiotic prophylaxis for orthognathic surgery: a prospective, randomized, double-blind clinical study. Journal of Oral and Maxillofacial Surgery.

